# Visualizing a Lung Opacity Behind Pleural Effusion Using Dynamic Chest Radiography

**DOI:** 10.1002/rcr2.70797

**Published:** 2026-10-29

**Authors:** Hiroki Ichikawa, Takuo Yoshida

**Affiliations:** ^1^ Division of Intensive Care, Department of Emergency and Disaster Medicine, Kashiwa Hospital The Jikei University School of Medicine Kashiwa Chiba Japan

**Keywords:** atelectasis, critical care, dynamic chest radiography, pleural effusion, portable radiography

## Abstract

Dynamic chest radiography with bone suppression and pixel‐value change analysis delineated a parenchymal abnormality obscured by pleural effusion on conventional portable chest radiography. This case demonstrates the visualization of a subtle lung abnormality using post‐processed dynamic chest radiography.

## Graphical abstract

Dynamic chest radiography with bone suppression and pixel‐value change analysis improved visualization of a parenchymal abnormality obscured by pleural effusion on conventional portable chest radiography. This case illustrates the potential of post‐processed dynamic chest radiography to reveal otherwise inconspicuous lung abnormalities at the bedside.

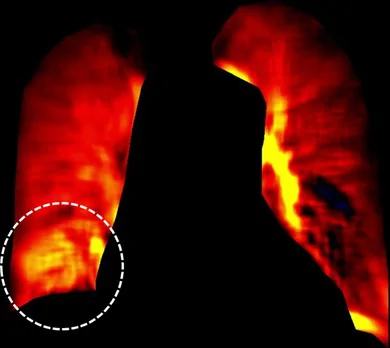

A 73‐year‐old man receiving high‐flow nasal cannula therapy underwent bedside dynamic chest radiography during intensive care. Computed tomography demonstrated a right pleural effusion with an underlying parenchymal abnormality, considered atelectasis or consolidation (Figure 1A). On conventional portable chest radiography, the pleural effusion was readily recognized, whereas the precise location of the underlying lung abnormality was difficult to delineate (Figure 1B).

**FIGURE 1:**
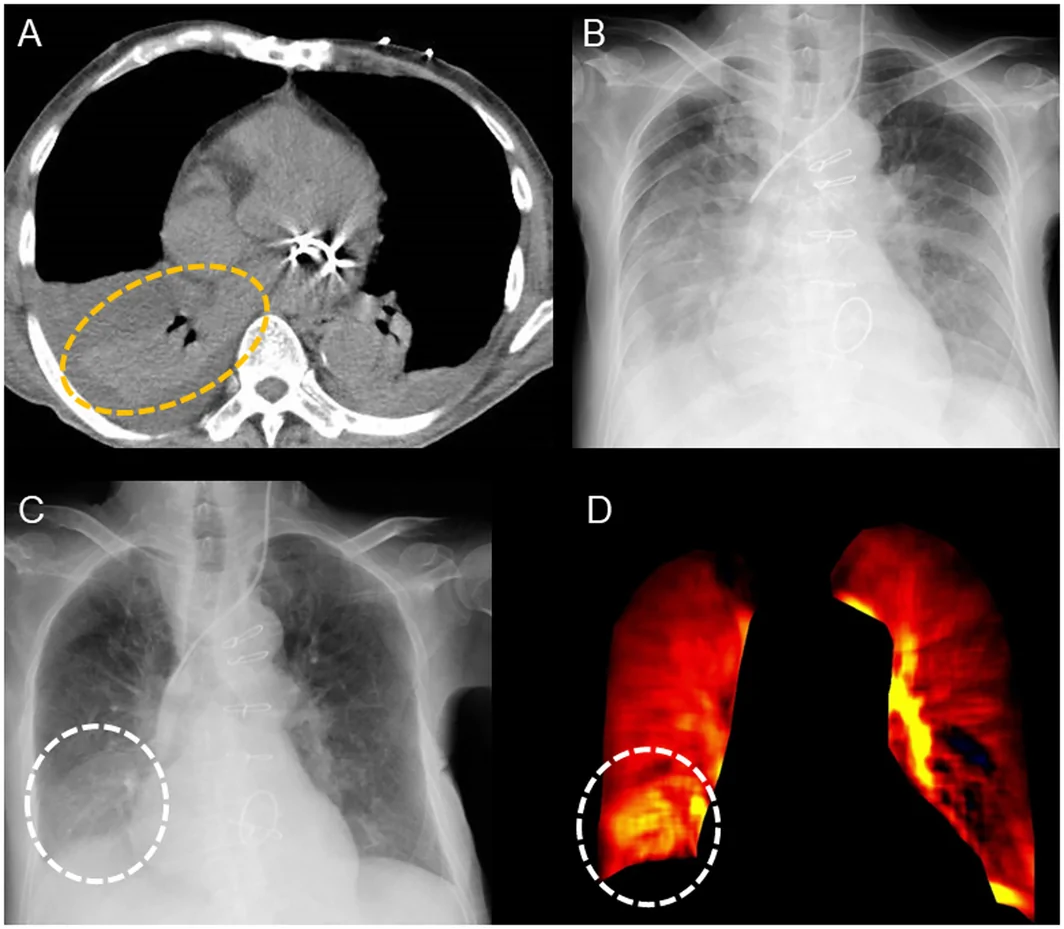
Dynamic chest radiography in pleural effusion–associated parenchymal abnormality. (A) Chest computed tomography showing a right pleural effusion with an underlying parenchymal abnormality, considered atelectasis or consolidation; the yellow circle indicates the abnormal region. (B) Conventional portable chest radiograph showing a right pleural effusion, with the underlying parenchymal abnormality poorly delineated. (C) Bone‐suppressed dynamic chest radiograph showing improved visualization of the abnormal density behind the pleural effusion; the white circle indicates the lesion. (D) Pixel‐value change image (PH2 mode) showing the abnormal region corresponding to the computed tomography finding; the white circle indicates the lesion.

Because pulmonary embolism was also considered after ICU admission but transport for contrast‐enhanced computed tomography was undesirable due to unstable respiratory status, dynamic chest radiography was performed as an additional bedside assessment. Bone‐suppressed imaging improved visualization of the abnormal density by reducing rib shadows and allowing inspiratory–expiratory comparison (Figure 1C and Video 1). Pixel‐value change analysis, which visualizes cardiac‐synchronous changes in lung‐field pixel values, clearly delineated the abnormal region corresponding to the computed tomography findings (Figure 1D and Video 2) [1]. The prominent PH2‐mode signal may represent apparent perfusion‐related enhancement caused by increased pulmonary vessel density per unit image area, rather than true hyperperfusion.

**VIDEO 1:** Bone‐suppressed dynamic chest radiography. Bone‐suppressed dynamic chest radiography showing improved visualization of the abnormal density behind the right pleural effusion. The white circle indicates the lesion. Video content can be viewed at https://onlinelibrary.wiley.com/doi/10.1002/rcr2.70797.

**VIDEO 2:** Pixel‐value change analysis (PH2 mode). Pixel‐value change analysis showing the abnormal region corresponding to the computed tomography finding behind the right pleural effusion. The white circle indicates the lesion. Video content can be viewed at https://onlinelibrary.wiley.com/doi/10.1002/rcr2.70797.

Dynamic chest radiography provides multiple post‐processed images from a single bedside acquisition [2]. This case illustrates how such post‐processing can make an otherwise inconspicuous parenchymal abnormality more apparent without requiring patient transport.

## Author Contributions

**Hiroki Ichikawa:** conceptualization, writing – original draft, methodology, investigation, visualization. **Takuo Yoshida:** conceptualization, investigation, methodology, writing – review and editing, visualization, supervision.

## Funding

The authors have nothing to report.

## Consent

The authors declare that written informed consent was obtained for the publication of this manuscript and accompanying images and attest that the form used to obtain consent from the patient complies with the Journal requirements as outlined in the author guidelines.

## Conflicts of Interest

The authors declare no conflicts of interest.

## Data Availability

Data sharing not applicable to this article as no datasets were generated or analysed during the current study.
